# Supplementary material for: Effects of miniaturization in the anatomy of the minute springtail Mesaphorura sylvatica (Hexapoda: Collembola: Tullbergiidae)
Source: PeerJ. 2019 Nov 13;7:e8037. doi: 10.7717/peerj.8037 (PMC6858819; doi:10.7717/peerj.8037)
Supplement: Table S1 [file peerj-07-8037-s001.pdf]

Table S1. Sizes of collembolan species with studied anatomy.

Species	Studied by	Modern name	Body size	Source of body size	Comments
Anurida maritima	Fernald (1890), Willem (1900), Lécaillon (1902), Denis (1928), Wolter (1963)	*Anurida maritima*	3 mm	Fjellberg, 1998	
Isotoma grisea	Prowazek (1900)	*Desoria* sp.	~1.8 mm	Fjellberg, 1998	Isotoma grisea is called *Desoria* today. This species has a strict taxonomical understanding, while in the past its name was applied to a wide group of species.
Achorutes viaticus	Prowazek (1900), Willem (1900)	*Hypogastrura viatica*	1.9 mm	Fjellberg, 1998	
Onychiurus fimetarius	Denis (1928)	*Onychiurus (sensu lato)* sp.	>1.3 mm	Gisin, 1960	In older publications the identification of this species is uncertain. Any species with complex PAO and without anal spines could be named as *O. fimetarius*. A very approximate average size is given.
Tomocerus catalanus	Denis (1928)	*Tomocerus catalanus*	4.5 mm	Denis, 1924	
Protanura carpenteri	Mukerji (1932)	*Protanura carpenteri*	1.2 to 2.5 mm	Mukerji, 1932	

Orchesella cincta	Folsom (1899), Bretfeld, (1963), Verhoef et al. (1979)	*Orchesella cincta*	3 mm	Fjellberg, 1998	
Neanura muscorum	Bretfeld, (1963), Wolter (1963)	*Neanura muscorum*	3.5 mm	Fjellberg, 1998	
Tomocerus longicornis	Lubbock, (1873)	*Pogonognathellus longicornis*	4-5 mm	Fjellberg, 1998	
Orchesella villosa	Imms (1939), Dallai et al. (2008)	*Orchesella villosa*	4 mm	Fjellberg, 1998	
Isotomurus palustris	Imms (1939)	*Isotomurus* sp.	2.5 mm	Fjellberg, 1998	In older publications the identification of this species is uncertain. The average size for the genus is given.
Podura aquatica	Willem (1900), Imms (1939)	*Podura aquatica*	2 mm	Fjellberg, 1998	
Sminthurus viridis	Davies (1927), Imms (1939)	*Sminthurus viridis*	3 mm	Fjellberg, 1998	
Tomocerus flavescens	Wolter (1963), Humbert (1979)	*Pogonognathellus flavescens*	4-5 mm	Fjellberg, 1998	
Friesea mirabilis	Wolter (1963)	*Friesea mirabilis*	1.9 mm	Fjellberg, 1998	
Brachystomella parvula	Wolter (1963)	*Brachystomella parvula*	1.0 mm	Fjellberg, 1998	
Odontella armata	Wolter (1963)	*Xenyllodes armatus*	1.0 mm	Fjellberg, 1998	
Sminthurus fuscus	Willem & Sabbe (1897), Willem (1900)	*Allacma fusca*	3-4 mm	Fjellberg, 1998	
Onychiurus quadriocellatus	Altner (1968)	*Protaphorura quadriocellata*	2.2 mm	Fjellberg, 1998	

Tomocerus minor	Humbert (1975)	*Tomocerus minor*	4 mm	Fjellberg, 1998	
Lepidocyrtus curvicollis	Humbert (1975)	*Lepidocyrtus curvicollis*	3 mm	Fjellberg, 1998	
Orchesella rufescens	Philiptschenko (1907)	*Orchesella spectabilis*	4 mm	Fjellberg, 1998	*O.rufescens* is a junior synonym of *O.spectabilis* (collembola.org.).
Folsomia candida	Kollmann et al. (2011)	*Folsomia candida*	2.5 mm	Fjellberg, 1998	
Protaphorura armata	Kollmann et al. (2011)	*Protaphorura armata*	1.8 mm	Fjellberg, 1998	
Tetrodontophora bielanensis	Kollmann et al. (2011)	*Tetrodontophora bielanensis*	5-9 mm	Gisin, 1960	
Allacma fusca	Dallai et al. (2000)	*Allacma fusca*	3-4 mm	Fjellberg, 1998	
Anurophorus laricis	Willem (1900), Lécaillon (1902)	*Anurophorus* sp.	1.4 mm	Fjellberg, 1998	In older publications the identification of this species is uncertain. The average size for the genus is given.
Lipura armata	Willem (1900)	*Protaphorura* sp.	1.8 mm		In older publications the identification of this species is uncertain. The average size for the genus is given.
Anura muscorum	Willem (1900)	*Neanura muscorum*	3.5 mm	Fjellberg, 1998	
Isotoma viridis	Willem (1900)	*Isotoma viridis*	3-4 mm	Fjellberg, 1998	
Tomocerus plumbeus	Willem (1900)	*Pogonognathellus flavescens*	4-5 mm	Fjellberg, 1998	So far *Tomocerus plumbeus* is considered to be a synonym of *P.flavescens* .
Papirius sp.	Willem (1900)	*Dicyrtomidae* g.sp.	2 mm	Fjellberg, 1998	The average size for the family is given.
Megalothorax minimus	Willem (1900)	*Megalothorax* sp.	0.5 mm	Schneider et d'Haese, 2013	In older publications the identification of this species is uncertain. The average size for the genus is given.

Additional reference

Denis, JR. 1924. Sur la faune française des Atérigotes, V. Note préliminaire. *Bulletin de la Société Entomologique de France* , 49:197–199

Fjellberg A. 1998. The Collembola of Fennoscandia and Denmark. Part 1: Poduromorpha fauna. *Entomologica Scandinavica* . Leiden:Brill Academic

Gisin H. 1960. Collembolanfauna Europas. *Museum Histoire Naturelle.* Genève, Switzerland

Schneider C, D´Haese CA. 2013. Morphological and molecular insights on Megalothorax: the largest Neelipleona genus revisited (Collembola). *Invertebrate Systematics*, 27:317–364
