# Supplementary material for: Effects of miniaturization in the anatomy of the minute springtail Mesaphorura sylvatica (Hexapoda: Collembola: Tullbergiidae)
Source: PeerJ. 2019 Nov 13;7:e8037. doi: 10.7717/peerj.8037 (PMC6858819; doi:10.7717/peerj.8037)
Supplement: Table S2 [file peerj-07-8037-s002.pdf]

Table S2. Volumes of organs of *Mesaphorura sylvatica*.

System	Volume, nl	Relative volume, %
Skeleton	0.048	5.8
Digestive and excretory systems	0.068	8.6
Central nervous system*	0.051	6.3
*of which the brain	0.016	2.2
Muscular system	0.038	5.2
Reproductive system	0.15	18.9
Circulatory system and fat body	0.44	55.2
