# Supplementary material for: Effects of miniaturization in the anatomy of the minute springtail Mesaphorura sylvatica (Hexapoda: Collembola: Tullbergiidae)
Source: PeerJ. 2019 Nov 13;7:e8037. doi: 10.7717/peerj.8037 (PMC6858819; doi:10.7717/peerj.8037)
Supplement: Table S3 [file peerj-07-8037-s003.pdf]

Table S3. Nomenclature of muscles used in the present study and in others.

Head

**This study, *Mesaphorura***	**Folsom (1899), *Orchesella***
1(Oe)	dil. oe. x7
-	dil.phy. X4
-	dep.
-	1. rot. l.
-	2. abd.
3(Mn)	3. ret. rot.
4(Mn)	4. ret.
5(Mn)	5. pr't. l.
-	6. pr't. ms.
7(Mn)	7. rot.
8(Mn)	8. rot.
9(Mn)	9. add.
10(Mn)	10. rot.
11(Mn)	-
12(Mn)	-
1(Mx)	1. ret. abd.
2(Mx)	2. add.
3(Mx)	3. add.
4(Mx)	4. add.
5(Mx)	5. pr't. add.
-	6. prt. add.
7(Mx)	7. add.
-	8. ret. add.
-	9. pr't. add.
-	10. add.
-	1. dep. p.
2(Lb)	2. lvt. ms.
-	3. dep. a.
4(Lb)	4. lvt. m.
5(Lb)	5. lvt. l.
dlm1	-
dlm2	-
1(An)	-

Thorax

**This study, *Mesaphorura***	**Bretfeld (1963), *Neanura***	**Bretfeld (1963), *Orchesella***
Prothorax	Prothorax	Prothorax
I dlm1	I dlm1	I dlm
I dlm2	I dlm2	I dlm
I vlm	I vlm	I vlm
I ism1	I ism1	I ism1
I ism2	I ism2	I ism2
-	-	I ism3
-	-	I ism4
I dvm1	I dvm1	-
I dvm2	I dvm2	-
-	I dvm3	-
-	I dvm4	-
-	I dvm5	-
-	I dvm6	-
I ldvm1	I ldvm1	I ldvm
dlm1 (in the head)	Lb dlm1	-
dlm2 (in the head)	Lb dlm2	-
-	Lb vlm1	Lb vlm1
-	Lb vlm2	Lb vlm2
-	-	Lb ism
Lb dvm1	Lb dvm1	-
Lb dvm2	Lb dvm2	-
-	Lb ldvm1	Lb ldvm?
-	Lb ldvm2	-
I scm1	-	-
I scm2	-	-
I scm3	-	-
I scm4	-	-
Mesothorax	Mesothorax	Mesothorax
II dlm1	II dlm1	II dlm1
II dlm2	II dlm2	II dlm2
II vlm	II vlm	II vlm
II ism1	II ism1	II ism1
II ism2	II ism2	II ism2
-	II ism3	II ism3
-	II ism4	II ism4
II dvm1	-	II dvm1
II dvm2	II dvm2	II dvm2
II dvm3	II dvm3	-
II dvm4	II dvm4	-
II dvm5	II dvm5	-
-	II dvm6	-
II ldvm1	II ldvm1	II ldvm1
II ldvm2	II ldvm2	II ldvm2
II ldvm3	II ldvm3	II ldvm3
-	II ldvm4	II ldvm4
II scm1	-	-
II scm2	-	-
II scm3	-	-
II scm4	-	-
II scm5	-	-
II scm6	-	-
Metathorax	Metathorax	Metathorax
III dlm1	III dlm1	III dlm1
III dlm2	III dlm2	III dlm2
III vlm	III vlm	III vlm
III ism1	III ism1	III ism1
III ism2	III ism2	III ism2
III dvm1	III dvm1	III dvm1
III dvm2	III dvm2	III dvm2
III dvm3	III dvm3	-
III dvm4	III dvm4	-
III dvm5	III dvm5	-
-	III dvm6	-
III ldvm1	III ldvm1	III ldvm1
III ldvm2	III ldvm2	III ldvm2
III ldvm3	III ldvm3	III ldvm3
-	III ldvm4	III ldvm4
III scm2	-	-
III scm3	-	-
III scm5	-	-
III scm6	-	-
III scm7	-	-

Abdomen

**This study, *Mesaphorura***	**Bretfeld (1963), *Neanura***	**Eisenbeis (1976), *Tomocerus***
A I dlm1	I dlm1	-
AI dlm2	I dlm2	-
AI vlm	I vlm	-
AI ism1	I ism1	-
AI ism2	I ism2	-
AI dvm1	I dvm1	-
AI dvm2	I dvm2	-
AI dvm3	I dvm3?	-
AI dvm4	I dvm4?	-
AI dvm VT	I dvm VT	-
AI lm	-	lm
AI pm1	-	pm1
AI pm2	-	pm2
AI pm3	-	pm3
AI pm4	-	pm4? pm5? pm6?
AI dm1	-	dm1
AI dm2	-	dm2
AI dm3	-	dm3
AI dm4	-	dm4
AI dm5	-	dm5? dm6?
AI ldvm1	I ldvm1	-
AII dlm1	II dlm1	-
AII dlm2	II dlm2	-
AII vlm	II vlm	-
AII ism1	II ism1	-
AII ism2	II ism2	-
AII dvm1	II dvm1	-
AII dvm2	II dvm2	-
AII dvm3	II dvm3	-
AII dvm4	II dvm4	-
AII ldvm1	II ldvm1	-
AII trm1	II trm1	-
AIII dlm1	III dlm1	-
AIII dlm2	III dlm2	-
AIII ism1	III ism1	-
AIII ism2	III ism2	-
AIII dvm1	III dvm1	-
AIII dvm2	III dvm2	-
AIII dvm3	III dvm3?	-
AIII dvm4	III dvm4?	-
AIII ldvm1	III ldvm1	-
AIV dlm1	IV dlm1	-
AIV dlm2	IV dlm2	-
AIV vlm	IV vlm	-
AIV ism1	IV ism1? IV ism2?	-
AIV dvm1	IV dvm1	-
AIV dvm2	IV dvm2	-
AIV dvm3	-	-
AIV ldvm3	IV ldvm3	-
AIV ldvm4	IV ldvm4	-
AIV ldvm5	IV ldvm5	-
AIV ldvm7	IV ldvm7	-
AV dlm1	V dlm1	-
AV dlm2	V dlm2	-
AV ism1	V ism1? V ism2?	-
AV ldvm1	-	-
AVl sm1	-	-
AVI sm2	-	-
AVl dvm1	-	-
AVI dvm2	-	-
AVI dvm3	-	-
AVl dvm4	-	-
