# Supplementary material for: Effects of miniaturization in the anatomy of the minute springtail Mesaphorura sylvatica (Hexapoda: Collembola: Tullbergiidae)
Source: PeerJ. 2019 Nov 13;7:e8037. doi: 10.7717/peerj.8037 (PMC6858819; doi:10.7717/peerj.8037)
Supplement: Figure S1 — An —antenna, bp —body of pseudotentorium, cer —cerebrum, cx1, 2, 3 —pro-, meso-, and metacoxae, eg —ovary lobe with eggs, gg1, gg1, 2, 3+ag —pro-, meso-, and metathoracic +abdominal ganglia, hp —hypopharynx, mg —midgut, Mn —mandible, Mx —maxilla, oes —oesophagus, rt —rectum, soeg —supraoesophagal ganglion, VT —ventral tube. [file peerj-07-8037-s004.pdf]

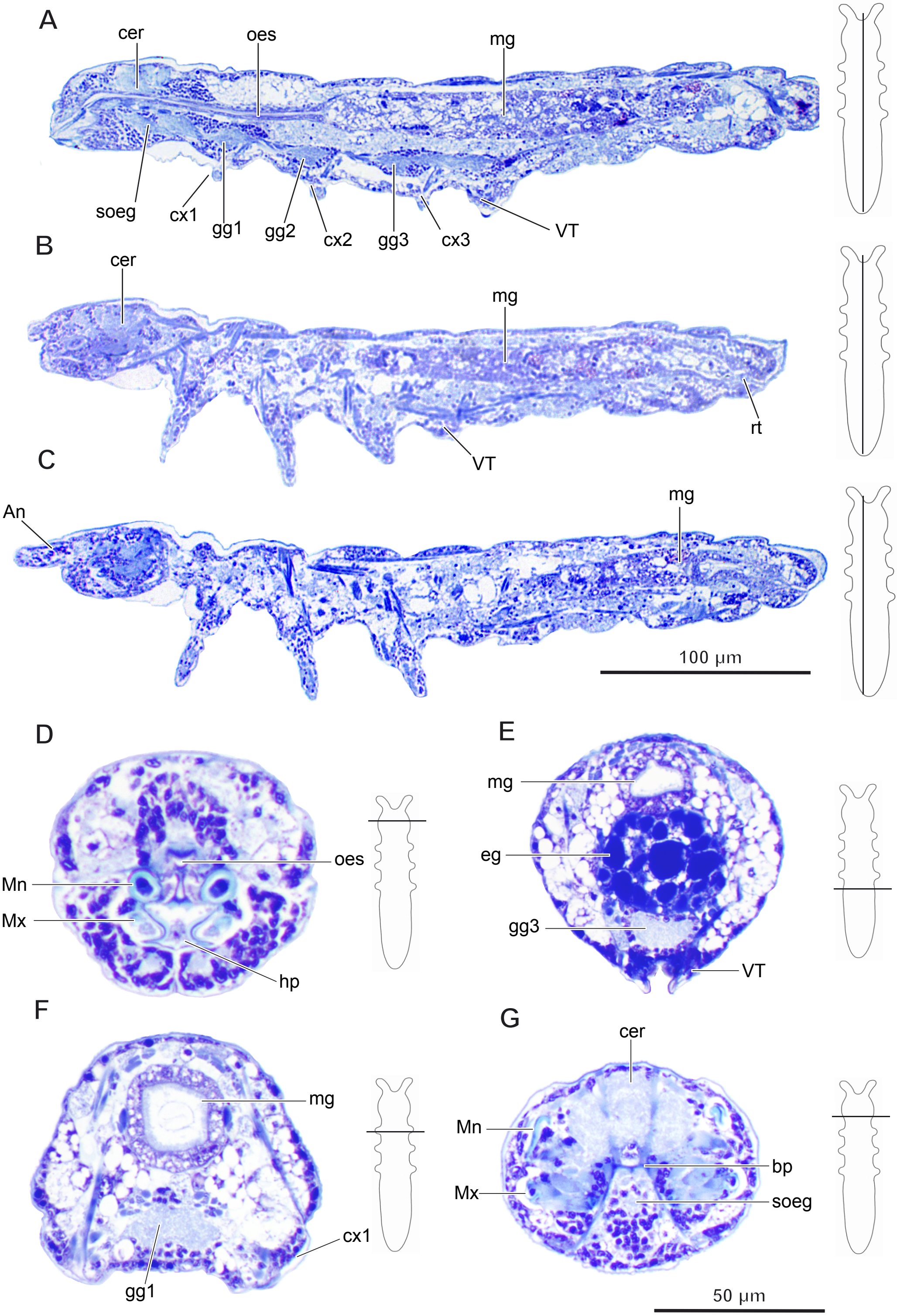

Fig. S1. Longitudinal (A – C) and cross-sections (D – G) of *Mesaphorura sylvatica*. An — antenna, bp — body of pseudotentorium, cer — cerebrum, cx1, 2, 3 — pro-, meso-, and metacoxae, eg — ovary lobe with eggs, gg1, gg1, 2, 3+ag — pro-, meso-, and metathoracic +abdominal ganglia, hp — hypopharynx, mg — midgut, Mn — mandible, Mx — maxilla, oes — oesophagus, rt — rectum, soeg — supraoesophagal ganglion, VT — ventral tube.
