# Supplementary material for: Effects of miniaturization in the anatomy of the minute springtail Mesaphorura sylvatica (Hexapoda: Collembola: Tullbergiidae)
Source: PeerJ. 2019 Nov 13;7:e8037. doi: 10.7717/peerj.8037 (PMC6858819; doi:10.7717/peerj.8037)
Supplement: Figure S2 — Click on the figure to start interactive 3D view. Colors: blue —cuticle, light blue —tentorium, green —digestive system, yellow —central nervous system, brown —musculature, purple —reproductive system, dark violet —excretory system. [file peerj-07-8037-s005.pdf]

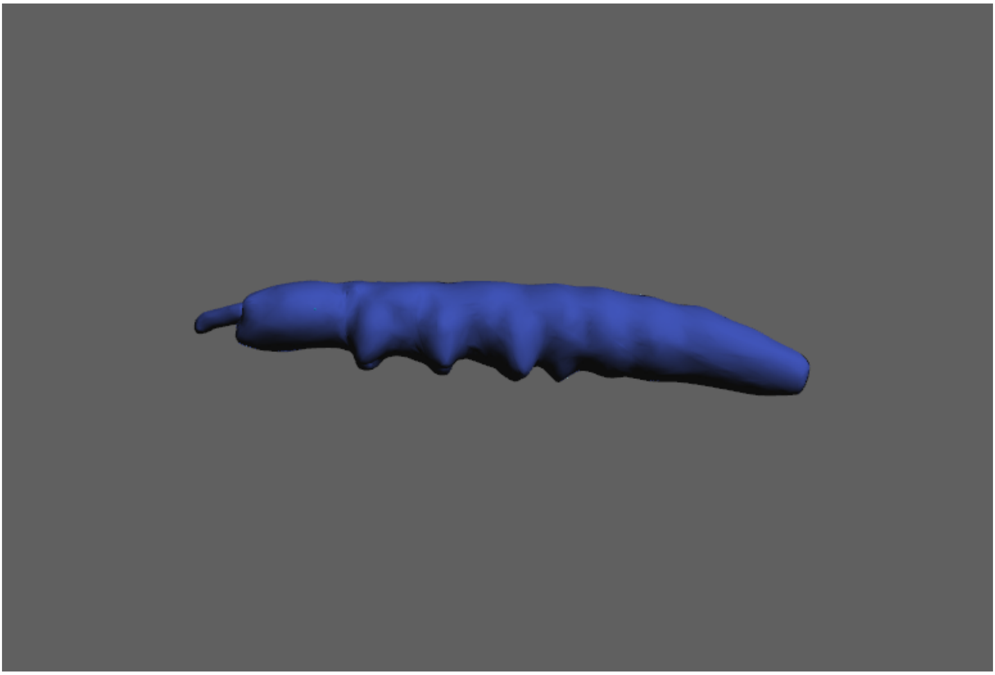

Fig. S2. Interactive animated 3D reconstruction of the *Mesaphorura sylvatica*. Click on the figure to start interactive 3D view. Colors: blue — cuticle, light blue — tentorium, green — digestive system, yellow — central nervous system, brown — musculature, purple — reproductive system, dark violet — excretory system.
